# Acceleration of Wound Healing in Rats by Modified Lignocellulose Based Sponge Containing Pentoxifylline Loaded Lecithin/Chitosan Nanoparticles

**DOI:** 10.3390/gels8100658

**Published:** 2022-10-15

**Authors:** Pouya Dehghani, Aliakbar Akbari, Milad Saadatkish, Jaleh Varshosaz, Monireh Kouhi, Mahdi Bodaghi

**Affiliations:** 1 Pharmacy Student’s Research Committee, School of Pharmacy, Isfahan University of Medical Sciences, Isfahan 81746-73461, Iran; 2 Novel Drug Delivery Systems Research Center, Department of Pharmaceutics, Faculty of Pharmacy, Isfahan University of Medical Sciences, Isfahan 81746-73461, Iran; 3 Dental Materials Research Center, Dental Research Institute, School of Dentistry, Isfahan University of Medical Sciences, Isfahan 81746-73461, Iran; 4 Department of Engineering, School of Science and Technology, Nottingham Trent University, Nottingham NG11 8NS, UK

**Keywords:** modified lignocellulose, pentoxifylline, hydrogels, nanoparticles, rat model, wound dressing

## Abstract

Dressing wounds accelerates the re-epithelialization process and changes the inflammatory environment towards healing. In the current study, a lignocellulose sponge containing pentoxifylline (PTX)-loaded lecithin/chitosan nanoparticles (LCNs) was developed to enhance the wound healing rate. Lecithin/chitosan nanoparticles were obtained by the solvent-injection method and characterized in terms of morphology, particle size distribution, and zeta potential. The lignocellulose hydrogels were functionalized through oxidation/amination and freeze-dried to obtain sponges. The prepared sponge was then loaded with LCNs/PTX to control drug release. The nanoparticle containing sponges were characterized using FTIR and SEM analysis. The drug release study from both nanoparticles and sponges was performed in PBS at 37 °C at different time points. The results demonstrated that PTX has sustained release from lignocellulose hydrogels. The wound healing was examined using a standard rat model. The results exhibited that PTX loaded hydrogels could achieve significantly accelerated and enhanced healing compared to the drug free hydrogels and the normal saline treatment. Histological examination of the healed skin confirmed the visual observations. Overall speaking, the in vivo assessment of the developed sponge asserts its suitability as wound dressing for treatment of chronic skin wounds.

## 1. Introduction

**Figure 1:**
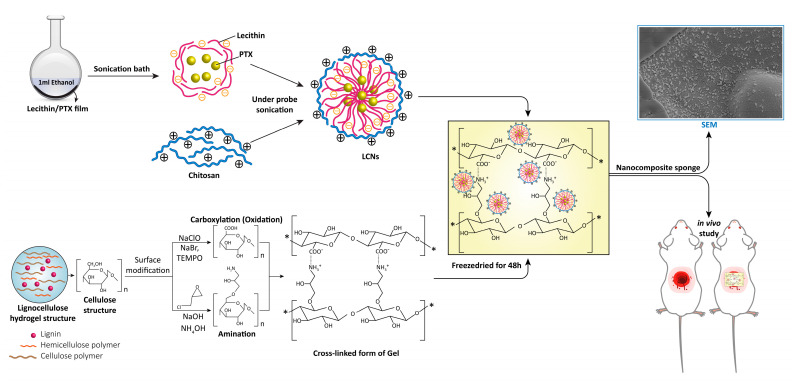
A schematic diagram representing the experimental design.

Design and production of an effective wound dressing capable of improving the wound healing process is a major biomedical challenge [1,2,3]. An ideal wound dressing is required to maintain the wound moisture, protect the wound area from infection and injury, absorb exudate, and reduce wound pain. Moreover, it should carry wound healing factors such as growth factors or nitric oxide to enhance the healing process [1,4]. Conventional wound dressings such as cotton wool, bandage, lint, or gauze do not offer these properties sufficiently. Recently, modern wound dressings such as hydrogels, cellulose sponges, and nanofibers have been investigated to improve the wound healing process [5,6]. The selection of suitable materials is crucial in designing wound dressing since functions and properties of dressings are mainly determined by the dressing materials. Lignocellulose (LC), as the naturally most abundant and renewable resource with unique properties such as excellent mechanical performance, biocompatibility, biodegradability, and multiple functional group, is a proper material for the development of dressings [7,8]. However, due to its relatively poor water solubility, its application in the biomedical field is limited. The physical and chemical modification of LC is an effective way to improve its physicochemical and biological properties [9,10]. It was reported that surface modification of cellulose materials through oxidation and amination can affect the cell adhesion and cellular uptake due to the favorable electrostatic interaction [11,12]. Moreover, it can produce a hydrogel with ionic strength-responsive swelling properties capable of controlled drug release after changing the conditions [13]. Pentoxifylline (PTX), an anti-inflammatory drug, is a synthetic methyl xantine [14]. It was reported that PTX can increase the wound healing rate by modulating gene expression of MMP-1, MMP-3, and TIMP-1 in normoglycemic rats [15]. It can be applied orally or intravenously; however, there are some disadvantages, such as several side effects related to the gastrointestinal tract and in the central nervous system. The topical administration of PTX could be an alternative to heal skin disorders; however, the predominantly hydrophilic nature of the drug makes it difficult for it to penetrate the skin [16]. Thus, encapsulation of PTX into a colloidal delivery system would offer reduced side effects, frequency of administration, and increased bioavailability [17]. In the present study, to control PTX release, it was encapsulated in lecithin/chitosan nanoparticles (LCNs). It was shown that LCNs are highly effective at delivering therapeutic agents transdermally [18,19]. LCNs have the synergetic advantages of both lecithin and chitosan, resulting in increasing the drug retention time at the target site and enhancing penetration of the drug [20,21,22,23,24]. It was hypothesized that the inclusion of PTX loaded LCNs into lignocellulose hydrogels can produce a dressing with the ability to control the release capable of enhancing the wound healing process. In this study, the LC sponge was prepared by surface modification of LC hydrogels through carboxylation (oxidation) and amination following by freeze drying and was then filled with PTX loaded LCNs (Figure 1 shows the schematic illustration of experimental procedures). The physicochemical properties of the developed nanoparticles and sponge, as well as in vitro drug release and in vivo wound healing, were investigated to study the suitability of the developed sponge for wound healing applications. The results reveal that both the LCNs and LC sponge possess a controlled release of PTX. Moreover, the LC sponge containing PTX-loaded LCNs increased the rate of wound healing compared to the control group.

## 2. Results and Discussion

**Figure 2:**
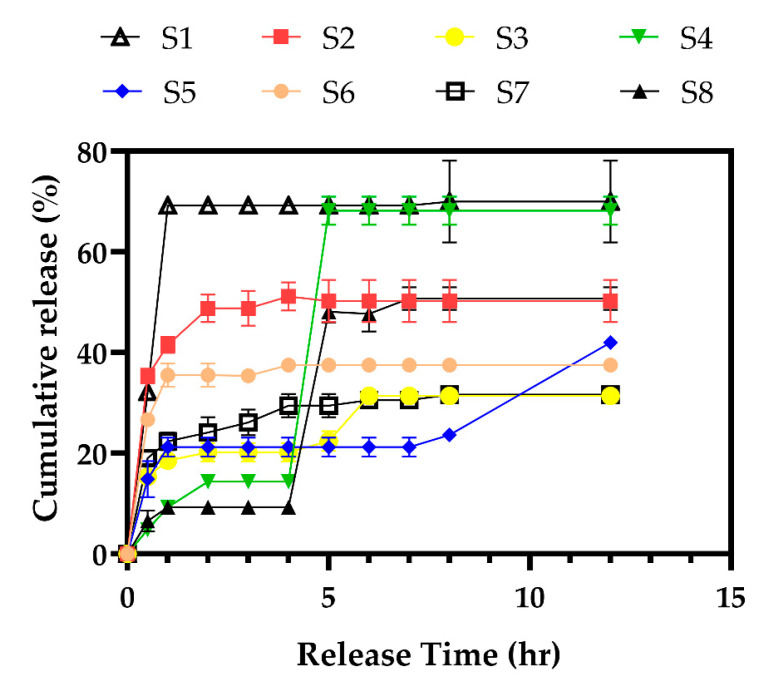
In vitro release profile of the PTX from different formulations of nanoparticles (the properties of nanoparticles and their formulations are summarized in Table 1).

LCNs have great potential for the transdermal delivery of therapeutic agents due to their ability to encapsulate drugs and sustain release [25]. In the current study, the LCNs nanoparticles were obtained by the injection of methanolic lecithin dispersion into a chitosan solution. Positively charged chitosan interacted with negatively charged lecithin through self-assembly. The content of LCNs and PTX-loaded LCNs and their characteristics, including size, polydispersity index (PDI), zeta potential and drug entrapment efficiency (EE), are summarized in Table 1, in which the obtained results are in the range of 259–869 nm, 0.042–0.373, 7.43–43.1 mV, and 30–62%, respectively. The low PDI of the nanoparticles indicates the uniform size distribution in all formulations. The drug release behavior from LCNs with different chitosan, lecithin, and PTX content during 12 h is plotted in Figure 2. Results show that at constant PTX and lecithin, the higher concentration of chitosan causes a lower release rate with a more controlled manner. Formulations with low levels of chitosan and lecithin (S1, S2) show a considerable burst release revealing the disability of the nanoparticle wall to protect fast release of the highly water soluble PTX. The burst release of PTX may also be related to the accumulation of the drug on the surface region of the nanoparticles.

**Figure 3:**
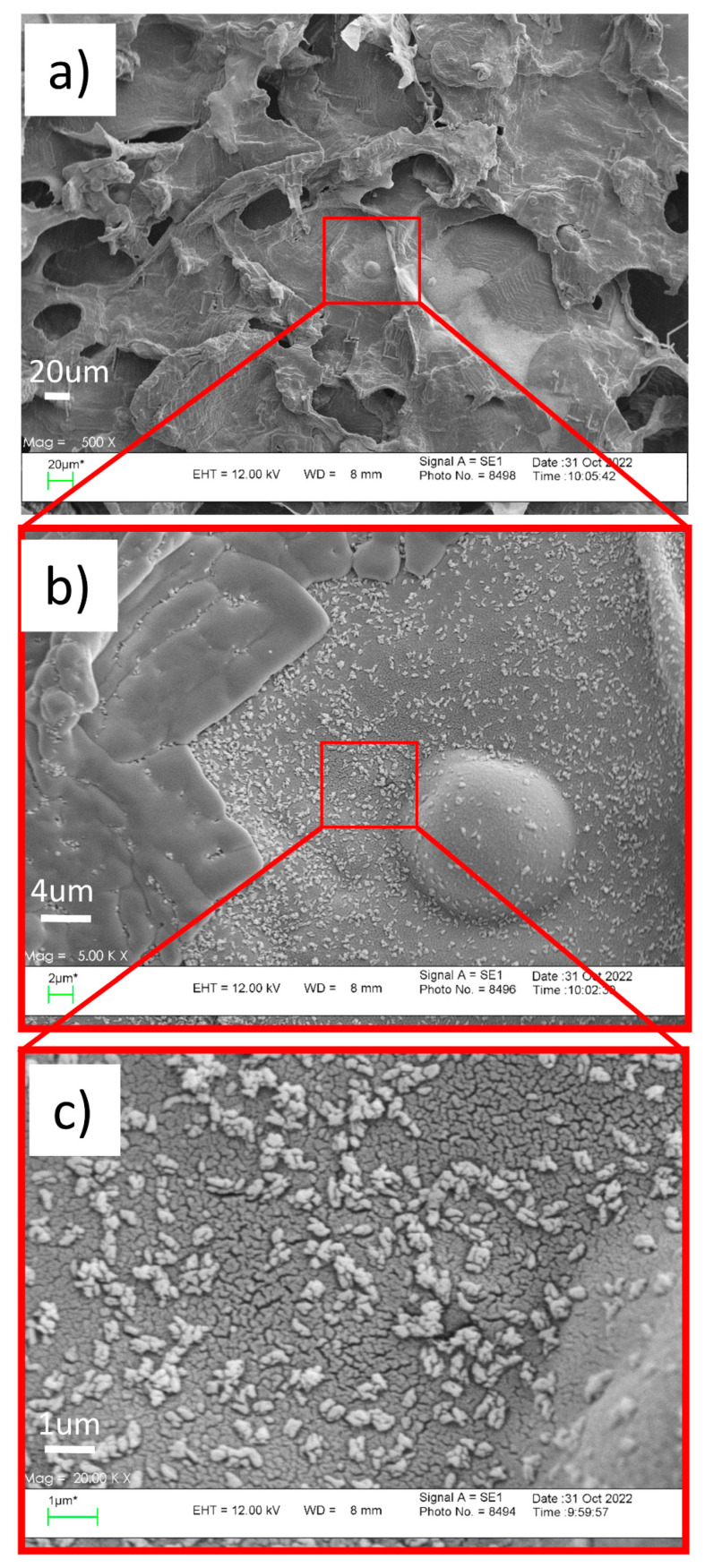
SEM micrograph of the LC sponge containing LCNs/PTX at (**a**) 500× and (**b**) 5000× and (**c**) 20,000×, (LCNs/PTX nanoparticles on the surface of sponge are more visible in the 20,000X image).

A full factorial design was used to optimize the process parameters for encapsulation of PTX in LCNs. The design parameters are the concentration of chitosan, lecithin, and PTX as their values are mentioned in Table 1. According to the results of Design Expert Software, the optimum formulation contained 1.9 mg/mL chitosan, 3.89 mg lecithin, and 1.89 PTX, which was predicted to have a particle size of 422.9 nm and drug entrapment of 43.5%. The proposed formulation was synthesized for the preparation of LC sponges containing LCNs/PTX. The morphologies of the optimized LCNs/PTX and prepared LC sponge are demonstrated in Figure 3. As can be seen in higher magnification images, the nanoparticles are entrapped successfully in the sponge pores. They have a uniform shape with obvious agglomeration.

**Figure 4:**
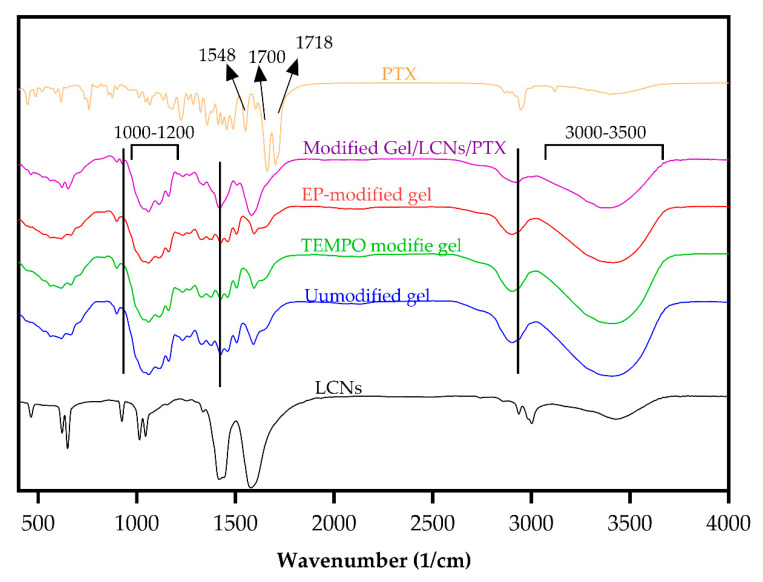
FTIR spectra of PTX, LCNs, unmodified hyhdrogels, TEMPO-modified hydrogels, EPI/TEMPO-modified hydrogels, and EPI/TEMPO-modified hydrogels containing LCNs/PTX.

FTIR spectroscopy analysis was performed to study the possible interaction of LC hydrogels with nanoparticles and the drug, as well as the effect of modification on the lignocellulose chemical bonds. Figure 4 illustrates the FTIR spectra of PTX, LCNs, unmodified hydrogels, TEMPO-modified hydrogels, epichlorohydrin (EPI)/TEMPO-modified hydrogels, and EPI/TEMPO modified hydrogels containing LCNs/PTX. In the FTIR spectra of PTX, peaks at 1700 cm^−1^, 1718 cm^−1^, 1548 cm^−1^, 756 cm^−1^, and 1412 cm^−1^ contribute to the C-O stretching vibration of the amide bond, the C-O stretching vibration of ketone, the N-H bending vibration of the amide, the C-N out of plane wagging of the amide, and the C-N stretching of the amide, respectively [26]. All TEMPO-modified hydrogels, EPI/TEMPO-modified hydrogels, and EPI/TEMPO modified hydrogels containing LCNs/PTX exhibit a broad absorption peak at 3000–3600 cm^−1^, 2900 cm^−1^, 1300–1400 cm^−1^, and 1000–1200 cm^−1^, which are attributed to O-H single bond stretching, C-H single bond stretching, O-H single bond and C-H single bond bending, and C–OH single bond and C-O single bond asymmetric stretching, respectively [27]. The peak at 1600 cm^−1^ is related to the C-O double bond stretching of carboxylate groups (COONa and COOCa) [28]. The O-H single bond peak intensity at 3000−3600 cm^−1^ reduces in EPI/TEMPO modified hydrogels, while the C-H single bond peak intensity at 1425 cm^−1^ increases in EPI/TEMPO modified hydrogels compared to TEMPO-modified hydrogels and unmodified hydrogels. These results clearly reveal the formation of covalent bonds between EPI and the hydroxyl groups [29].

**Figure 5:**
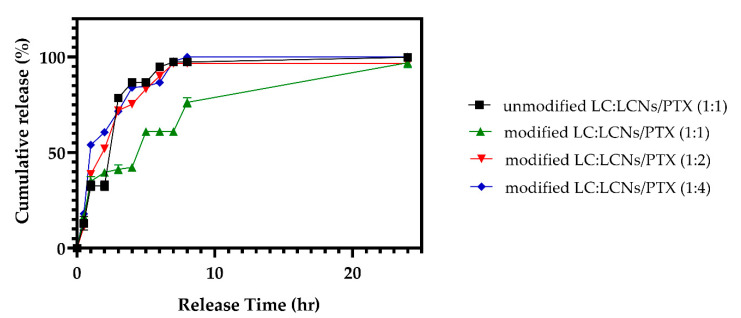
In vitro release profile of the PTX from unmodified hydrogels containing LCNs/PTX and modified hydrogels containing LCNs/PTX with different LC:LCNs/PTX ratios. Modified hydrogels containing lower concentrations of LCNs/PTX revealed a lower release rate compared to other samples.

Figure 5 depicts the PTX release curves from unmodified hydrogels containing LCNs/PTX and modified hydrogels containing LCNs/PTX with different LC/nanoparticle ratios. At a higher concentration of LCNs/PTX, the hydrogels show burst release of approximately 75% in the first 3 h. Among different samples, the LC:LCNs/PTX (1:1) sample with a lower nanoparticle concentration shows a more sustained release rate such that, in the first 9 h, 75% of the drug was released.

**Figure 6:**
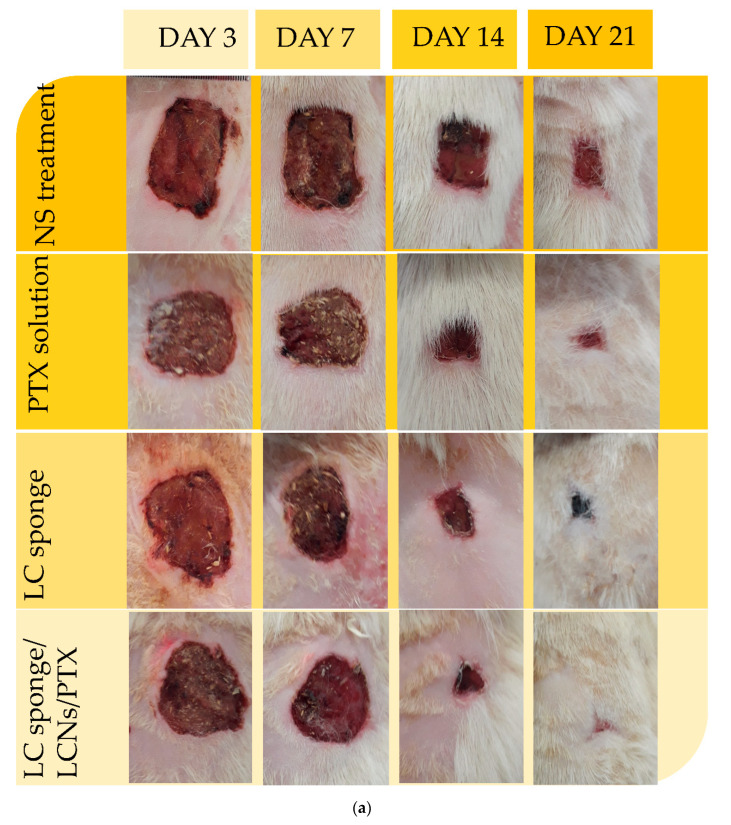

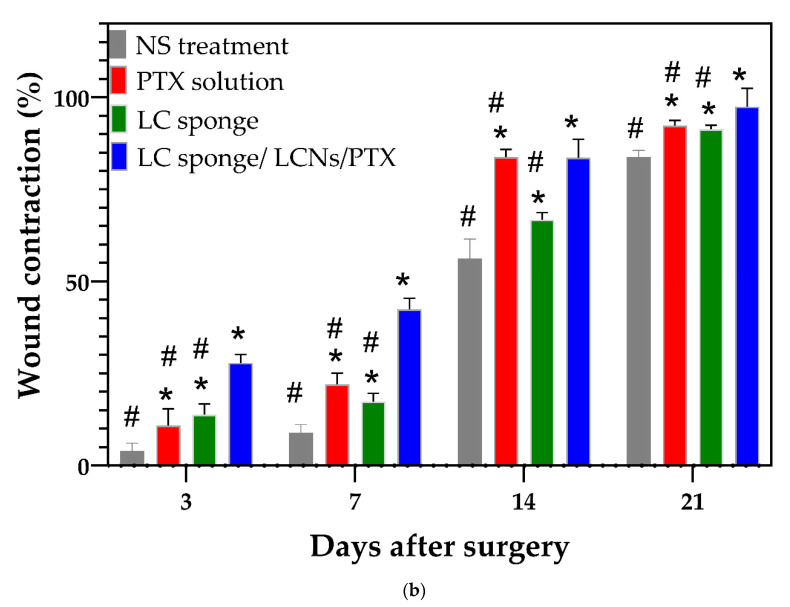
Wound healing process of treated rats with normal saline, free PTX, LC sponge, and LC sponge containing LCNs/PTX, (**a**) Photographical images of wound contraction on different days of control and treated animals. (**b**) Representation of percentage wound contraction of control and treated wounds. Values are expressed as mean ± SD for twenty-four animals. *: indicates the significant differences with NS treatment group and #: indicates the significant differences with LC sponge containing LCNs/PTX group.

In numerous studies, PTX was found to facilitate the wound healing process in a wide variety of pathological conditions, including ulcers in a venous leg, the syndrome of diabetes, wounds caused by physicochemical factors, and radiation-related injuries [30,31]. Similarly, hydrogel materials exhibited superiority over traditional wound treatment (ointment, etc.) due to their well-known properties of high biocompatibility, biodegradability, very low immunogenicity, excellent drug delivery (e.g., antibiotics), and ease of use. More importantly, treatment with the hydrogels led to a preservation of the breathability and good moistening of the tissue, which is due to the galenics of the gels consisting of water [32]. Furthermore, the promotion of re-vascularization, a significant lower infection rate due to moist wound management, less scarring, and aesthetically better healing results can be achieved by applying hydrogels in wound treatments [33]. Figure 5 demonstrates the wound healing process in different groups of NS treatment, PTX solution, LC sponge, and LC sponge containing LCNs/PTX, on days 3, 7, 14, and 21 after surgery. In all groups, the wound size reduces because of the body’s natural response and the biological proceeding of wound healing. Still, on the third and seventh days of the test, the size of the wound in the LC containing LCNs/PTX nanoparticles is obviously smaller than other groups. The results of the wound contraction measurement summarized in Figure 6 reveal that the size contraction from the PTX containing sponge was significantly larger than the other group on day 7 (*p* ≤ 0.05). On the 14th and 21st days, wound healing proceeded in the LCNs/PTX-loaded sponge group with almost the same initial speed; however, the healing rate of the wound in other groups decreased significantly (*p* < 0.05). In general, inner-group statistical comparison between wound size revealed that the wounds in the sponge group containing PTX significantly increased on days 14, 7, 3, and 21, respectively, 27.83 ± 2.83, 42.39 ± 2.99, 83.56 ± 5.03, and 97.44 ± 0.85, which was faster than other groups. By comparing the treatments with the developed hydrogels and normal saline, it was concluded that all the treatments developed in this study increased the wound healing rate compared to normal saline. Additionally, by comparing the two groups of PTX solution and LC sponge (without PTX), it can be seen that despite the better effect of PTX, there is no significant difference between the two groups.

**Figure 7:**
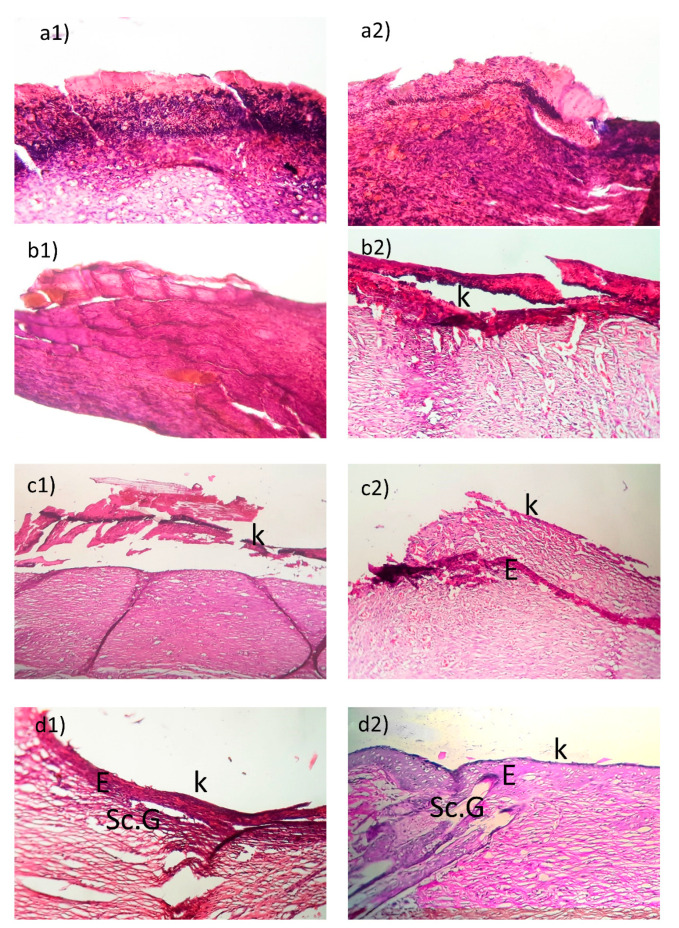
Histological changes during the wound-healing process. Histology of the wound tissue of experimental groups, (**a1**,**a2**) NS treatment, (**b1**,**b2**) PTX solution, (**c1**,**c2**) LC sponge and (**d1**,**d2**) LC sponge containing LCNs/PTX. N.F, follicles; Sc. G, sebaceous glands; K, keratin layer; E, epidermis layer.

According to the microscopic observations, aseptic conditions were confirmed to be in all the wounds. As shown in Figure 7, in wounds treated with normal saline, the inflammatory response was higher than the other treated wounds. Necrotic debris on the surface of the tissue was almost removed in the wound treated with the LC sponge containing LCNs/PTX. As observed in H&E staining images, the areas associated with neovascularization (open lumen vascular structures and endothelial cell clusters) were greater in the blank group than other groups treated with wound dressings. The reduction of these areas in LC sponge containing LCNs/PTX wound dressings groups indicates the completion of the healing process. Other signs of wound healing, in addition to neovascularization areas visible in this group, include the thickness of the epidermis layer (E) and the formation of the keratin layer (K) on the epidermis layer, as well as the presence of skin appendages such as new follicles (N.F) and sebaceous glands (Sc. G) in the dermis layer. These can be attributed to the role of PTX in facilitating tissue repair and the wound healing process. PTX activity can be divided into four main categories, including: (1) PTX can increase the deformation ability of red blood cells (RBCs), while decreasing the RBCs aggregation and vasoconstriction and has reducing effects on blood viscosity [34]. (2) PTX showed immunological activities that are effective by various mechanisms such as: inhibiting the activation of T and B lymphocytes, reducing the release of TNF-α from monocytes, releasing of peroxides from neutrophils, reducing the neutrophil degranulation, decreasing the leukocytes aggregation/ adhesion, and increasing their deformability and chemotaxis. PTX also modulates or blocks the inflammatory actions of IL-1 and TNF-α on neutrophils and has some effects on other cytokines, such as IL-6, IL-8, VEGF, and TGF-β1 [15,35,36] (3). Effects on platelet aggregation and adhesion [37], and finally (4), effects on properties of connective tissue and direct wound healing such as decreasing the levels of fibroblast collagen, fibronectin, and glycosaminoglycans, as well as reducing the response of fibroblast to IL-1 and TNF-α [38].

## 3. Conclusions

In this study, the EPI/TEMPO modified lignocellulose hydrogels were developed and filled with LCNs containing PTX to control drug release for wound healing applications. Using full factorial design software, an optimized formulation of nanoparticles prepared from different concentrations of ingredients was suggested based on the results of particle size, zeta potential, and drug load/release. The prepared nanoparticles were entrapped successfully in the sponge pores and attached to its pore wall, as is evident from SEM observation. The drug release study from hydrogels revealed that a lower concentration of PTX in the hydrogels resulted in more sustained drug release. The results of the wound healing evaluation in the animal model indicated the higher healing rate by treatment with sponges containing PTX-loaded LCNs. The developed sponge with control release behavior has shown the potential to be used in wound treatment applications.

## 4. Materials and Methods

### 4.1. Materials

PTX was kindly provided by Amin Pharmaceutical Company (Isfahan, Iran), soy lecithin (Degussa, GmbH, Freising, Germany), low molecular weight chitosan from Sigma Company (Saint Louis, MO, US), glacial acetic acid, and all other reagents were purchased from Merck Chemical Company (Darmstadt, Germany), LC nanofibrils hydrogel was purchased from Nano Novin Polymer (Gorgan, Iran).

### 4.2. Preparation of Chitosan Coated Lecithin Nanoparticles

In order to prepare drug-loaded lecithin nanoparticles, the desired amount of the drug was dissolved in methanol, followed by the dissolution of lecithin in the prepared drug solution using sonication. The methanol was evaporated using a rotary evaporator to obtain the PTX-loaded lecithin film. The prepared film was dispersed in 1 mL methanol using a sonication bath to re-disperse the drug-loaded lecithin films. The chitosan solution was prepared by dissolving defined amounts of chitosan (Table 1) in 5 mL distilled water containing 1% acetic acid for 24 h, and the pH was increased to 4.7 by adding NaOH 1M solution. For the preparation of chitosan-coated lecithin nanoparticles (LCNs/PTX), the methanolic lecithin–PTX solution was added to the chitosan solution under probe sonication. The blank nanoparticles were prepared using a similar procedure without the PTX addition. A full factorial design suggested by the Design Expert software (version 10.0.7, US) was used for optimization of the chitosan concentration (A), lecithin amount (B), and PTX amount (C) as main independent variables. NPs size, zeta potential, loading efficiency, and release efficiency were studied as dependent variables. The minimum and maximum levels of each parameter were determined based on preliminary experiments. The composition of prepared NPs and observed responses are presented in Table 1. Design Expert software was used to analyze the statistical significance and optimization of the NPs formulation.

### 4.3. Particle Size and Zeta Potential Measurement

First, 0.1 mg of prepared nanoparticles was dispersed in 10 mL of deionized water for 30 min in a sonication bath (85 W, 42 kHz), and their particle size, polydispersity index (PDI), and zeta potential were read using Zetasizer (Zetasizer 3600, Malvern Instrument Ltd., Worchestershire, UK).

### 4.4. Entrapment Efficiency

To determine the entrapment efficiency of the nanoparticles, the unentrapped PTX was measured by centrifuging a suspension of LCNs-PTX nanoparticles in deionized water at 12,000 rpm. The UV absorbance of the supernatant was read spectrophotometrically at 274 nm. Then, the entrapment efficiency (EE%) was calculated as:

EE % = (Initial drug quantity − Unentrapped quantity of drug)/(Total drug) × 100%.

### 4.5. Surface Modification of LC Hydrogels

The surface modification of LC hydrogels was performed through carboxylation (oxidation) and amination according to the previously reported method with some minor modification [13]. For the oxidation reaction, 3.145 g of LC hydrogels (equal to 0.1 g of dry weight) was suspended in an 80 mL solution containing 15 mg 2,2,6,6-tetramethylpiperidine-1-oxy (TEMPO) and 98.87 mg of sodium bromide. Then, 101 µL of sodium hypochlorite was added to start the oxidation reaction. The pH was kept at 10.5 by adding an appropriate amount of 1 M sodium hydroxide. The reaction was continued on the stirrer at room temperature and 600 rpm for 4 h. Finally, 2 mL of ethanol was added to the reaction mixture. The obtained hydrogel was washed with distilled water by centrifugation at 7800 rpm for 10 min and repeated until the oxidized hydrogel reached the pH of approximately 7. For the amination reaction, a 3.145 g sample of hydrogel was added to the 40 mL of 1 M sodium hydroxide solution and heated with constant stirring to 60 °C. Then, 140 µL of epichlorohydrin was added and allowed to react with stirring at 600 rpm and 60 °C for 2 h. The solution was washed with distilled water by centrifugation at 7800 rpm for approximately 10 min until the pH reached below 12. The precipitated hydrogel was re-suspended in 40 mL of 0.01 M sodium hydroxide, and the proper amount of ammonium hydroxide (29.4% *w/v*) was added to adjust the pH of the solution to 12. The mixture was then left to stir at 600 rpm and 60 °C for an additional 2 h. The resultant hydrogel was washed and centrifuged until the pH was reduced to 7 and stored at 4 °C.

### 4.6. Preparation of LC Sponge Containing Drug Loaded Nanoparticles

The cationic and anionic forms of the surface modified nanofibers were added together in equal amounts to obtain the cross-linked form hydrogel. To load nanoparticles in the prepared hydrogel, 2 mg of nanoparticles were dispersed in 1 mL of deionized water and mixed evenly with different amounts of the prepared hydrogels (0.5, 1, and 2 g). The mixtures were then frozen overnight and freeze dried for 48 h to obtain nanocomposite sponges. For comparison purposes, LCNs and LCNs/PTX nanoparticles were also added to 2 g of non-modified hydrogel and freeze dried in a similar manner to assess the effect of cross-linking on the release profile of drug.

### 4.7. Fourier Transform Infrared Spectroscopy

All samples including LCNs/PTX nanoparticles and sponges were ground well with potassium bromide and made into pellets. The spectra of samples were recorded using a FTIR spectrometer (JASCO model FT/IR 6300FV, Tokyo, Japan) in the 350–4000-cm^−1^ region.

### 4.8. SEM Observation

The morphologies of LCNs/PTX nanoparticles and dried hydrogels were studied using field emission scanning electron microscopy (FESEM-SEM Hitachi F41100, Japan) at an acceleration voltage of 20 kV.

### 4.9. In Vitro Drug Release Studies

Drug release studies were carried out using the dialysis technique, in which 0.5 mL of the drug/nanoparticles dispersion or 1.5–2 g sponge were placed inside a dialysis bag, sealed, and submerged in release media (19.5 mL of phosphate buffer saline solution and Tween 80 at pH 7.4) while stirred at 200 rpm. Through the dialysis membrane, the released drug diffused to the outer release media, from where the samples were taken for further analysis. To measure the amount of drug released, 1 mL of the release medium was centrifuged for 5 min at 12,000 rpm and UV-spectrophotometrically examined at 274 nm, and returned to the test media. The studies were repeated three times, and the results were reported as mean ± standard deviation.

### 4.10. In Vivo Wound Healing Studies

All experimental procedures were in accordance with approved guidelines of the Animal Committee of Isfahan University of Medical sciences, Iran (ethical code No.: IR.MUI.RESEARCH.REC.1397.267). A full-thickness excisional wound model was used to evaluate the wound healing ability of the prepared hydrogels. Twenty-four healthy adult male Wistar rats (8–10 months old, weighing 220–250 g) were obtained from the animal lab of the Department of Pharmaceutics, Isfahan University of Medical Sciences. The animals were kept at a controlled ad libitum condition of 22 ± 2 °C and 50–60% humidity. Then, the rats were randomly divided into four groups (6 rats in each group), including blank group, PTX group, PTX free sponge, PTX loaded sponge. The animals were anesthetized by intraperitoneal injection of 10 mg/kg xylazine and 80 mg/kg ketamine, then their backs were shaved, and an approximate 3 cm midline incision was made in the skin and subcutaneous tissue. The wound in blank group was treated with 1 mL of normal saline. In other groups the content of PTX was set to be 1.5 mg. The wound was covered with a gauze dressing and the dressing was changed every day.

#### 4.10.1. Macroscopic Observation of the Wound-Healing Process

After 3, 7, 14, and 21 days of wound creation, the healing progress was recorded using a digital camera. The rate of wound closure and reduction in the wound size were determined by measuring wound area using an image analyzing program. The wound closure was calculated as:

Wound closure (%) = [1 − (open wound area/initial wound area)] × 100

After the study period, the animals were sacrificed and skin tissue samples were removed surgically for histological evaluation.

#### 4.10.2. Histological Examination

On day 21 post-treatment, the treated excision wounds were immediately fixed in 10% formaldehyde, dehydrated with ethanol (70%), and then were embedded in paraffin blocks. The blocks were sectioned into 5 µm slides and stained with hematoxylin-eosin (H&E) to study the best stage of healing. Stained samples were examined under a light microscope (Olympus CX 21, Tokyo, Japan), and a digital camera was used to take the photo image from stained slides.

### 4.11. Statistical Analysis

The data were presented as mean and standard deviation. The results were statistically analyzed by IBM SPSS Statistics 26 Software using a one-way ANOVA test. In all of the evaluations, *p* < 0.05 was considered statistically significant.

## Figures and Tables

**Table 1:** The amount of chitosan, lecithin, and PTX in the developed nanoparticles, as well as properties of the developed nanoparticles, including size, polydispersity index, zeta potential, and drug entrapment efficiency.

Sample No	Chitosan (mg/mL)	Lecithin (mg)	PTX (mg)	Size (nm)	PDI	Zeta Potential (mv)	EE (%)
S1	1	2.5	1	321.4 ± 21.6	0.275	13.1 ± 0.8	35 ± 7.3
S2	1	2.5	2	259.3 ± 11	0.042	7.43 ± 0.35	47 ± 6.8
S3	1	5	1	329.3 ± 25.9	0.188	18.3 ± 2.1	30 ± 0.3
S4	1	5	2	508.2 ± 32	0.373	19 ± 2.2	45 ± 1.2
S5	2	2.5	1	864.6 ± 33.4	0.121	14.6 ± 0.5	43 ± 0.4
S6	2	2.5	2	306.1 ± 18.2	0.14	15.5 ± 1.1	41 ± 6.5
S7	2	5	1	365.2 ± 28.1	0.059	17.8 ± 1.8	62 ± 0.3
S8	2	5	2	429.4 ± 25.3	0.199	18.3 ± 0.09	45 ± 3.2

## Data Availability

All data are available on request.
